# Associations between maternal awakening salivary cortisol levels in mid-pregnancy and adverse birth outcomes

**DOI:** 10.1007/s00404-022-06513-4

**Published:** 2022-03-23

**Authors:** Richelle Vlenterie, Judith B. Prins, Nel Roeleveld, Marleen M. H. J. van Gelder

**Affiliations:** 1 Department for Health Evidence, Radboud Institute for Health Sciences, Radboud University Medical Center, Nijmegen, The Netherlands; 2 Department of Medical Psychology, Radboud Institute for Health Sciences, Radboud University Medical Center, Nijmegen, The Netherlands

**Keywords:** Hypothalamic-pituitary-adrenal (HPA) axis, Low birth weight, Preterm birth, PRIDE Study, Small-for-gestational age

## Abstract

**Purpose:**

Elevated levels of maternal cortisol have been hypothesized as the intermediate process between symptoms of depression and psychosocial stress during pregnancy and adverse birth outcomes. Therefore, we examined associations between cortisol levels in the second trimester of pregnancy and risks of three common birth outcomes in a nested case–control study.

**Methods:**

This study was embedded in the PRIDE Study (*n* = 3,019), from which we selected all cases with preterm birth (*n* = 64), low birth weight (*n* = 49), and small-for-gestational age (SGA; *n* = 65), and 260 randomly selected controls, among the participants who provided a single awakening saliva sample in approximately gestational week 19 in 2012–2016. Multivariable linear and logistic regression was performed to assess the associations between continuous and categorized cortisol levels and the selected outcomes.

**Results:**

We did not observe any associations between maternal cortisol levels and preterm birth and low birth weight. However, high cortisol levels (≥ 90th percentile) seemed to be associated with SGA (adjusted odds ratio 2.1, 95% confidence interval 0.9–4.8), in particular among girls (adjusted odds ratio 3.7, 95% confidence interval 1.1–11.9, based on eight exposed cases) in an exploratory analysis.

**Conclusion:**

The results of this study showed no suggestions of associations between maternal awakening cortisol levels in mid-pregnancy and adverse birth outcomes, except for an increased risk of SGA.

## Introduction

Common adverse birth outcomes, including preterm birth, low birth weight, and small-for-gestational age (SGA), are associated with neonatal mortality and long-term health problems, including neurodevelopmental impairments, respiratory and gastrointestinal complications, and higher sympathetic activity, which is considered a risk factor for cardiovascular disease [1–4]. Therefore, obtaining more insight into their etiology and identifying potential measures for prevention may lead to a great impact on public health. Previous research linked maternal psychosocial problems with increased risks of these birth outcomes, but the underlying mechanisms have not been fully understood yet [5]. One of the hypothesized intermediaries for this association is maternal cortisol, a glucocorticoid [6–8].

Cortisol is the end metabolite of the hypothalamic–pituitary–adrenal (HPA) axis and is essential in normal brain development [9]. Throughout pregnancy, maternal cortisol levels increase twofold, and cortisol crosses the placenta, accounting for 30–40% of the variability in fetal concentrations [10]. Psychosocial stress is hypothesized to dysregulate the HPA axis through hypersecretion of cortisol, but the results of previous studies among pregnant women are conflicting in this respect [11–13]. Other factors, including age, medical conditions, obesity, inflammation, physical inactivity, smoking, and alcohol use may also result in elevated cortisol levels [14–16].

Several biological mechanisms have been proposed for linking elevated maternal cortisol levels to infant birth weight. Fetal exposure to elevated cortisol levels may dysregulate fetal autonomic nervous system activity and result in a high degree of calorie expenditure by mobilizing fetal energy stores through glycogenesis [6]. Alternatively, cortisol combined with norepinephrine may induce uterine artery vasoconstriction, resulting in reduced uterine blood flow, restricting nutrient and oxygen supply to the fetus [17]. Cortisol could also affect birth weight by stimulating the production and release of placental corticotrophin releasing hormone (CRH), leading to shortened gestation [18]. The latter may also be affected by the bi-directional association between inflammation and cortisol [19].

The results of previous studies on the associations between elevated maternal cortisol, fetal growth, and gestational age at birth were inconsistent [7, 20–23]. Study design features explain at least part of the heterogeneity in findings. These include different sampling approaches (in blood, saliva, or hair) and variations in timing of sample collection (i.e. trimester of pregnancy) and time of day at sampling. Furthermore, many previous studies had small sample sizes and limited data on covariates, possibly resulting in residual confounding.

As preterm birth, low birth weight, and SGA occur frequently, more research is needed to understand the etiology of these adverse birth outcomes and the potential role of maternal elevated cortisol levels during pregnancy therein. Using the ‘Meet in the Middle’ approach [24, 25], we hypothesized that elevated maternal salivary cortisol levels in mid-pregnancy are associated with increased risks of preterm birth, low birth weight, and SGA. A nested case–control design embedded in a large prospective cohort study was applied to examine this hypothesis while adjusting for a range of potential confounders. Furthermore, we explored whether fetal sex affects the associations between maternal salivary cortisol levels and the selected adverse birth outcomes, as the maternal HPA axis varies according to the sex of the fetus [26–29], potentially leading to higher risks among female fetuses [30].

## Materials and methods

### General design PRIDE Study

This study was embedded in the PRegnancy and Infant DEvelopment (PRIDE) Study [31, 32], an ongoing prospective cohort study among Dutch women enrolled in early pregnancy. In short, pregnant women of 18 years of age and above, able to read and understand the Dutch language, and not more than 16 weeks pregnant were invited to participate in the PRIDE Study by their midwife or gynecologist at their first prenatal care visit. After providing informed consent, participants completed three web-based questionnaires during pregnancy, one questionnaire two months after the estimated date of delivery, and biannual questionnaires from six months post-partum onwards. Paper-based questionnaires were available for women who could not or did not want to participate through the Internet. The baseline questionnaire was administered around gestational weeks 8–12, the second questionnaire around gestational week 17, and the third questionnaire around gestational week 34. In these questionnaires, questions were asked about demographic factors, obstetric history, maternal health, pregnancy complications, lifestyle factors, current depression or a history of depression, and environmental and occupational exposures. The first postnatal questionnaire was focused on birth outcomes and the health of the infant. Furthermore, consent was asked for review of obstetric records. The PRIDE Study was approved by the Regional Committee on Research involving Human Subjects Arnhem-Nijmegen (CMO 2009/305).

### Cortisol collection and assay

Participants of the PRIDE Study were asked to optionally donate a single awakening saliva sample after completing the second prenatal questionnaire. Participants who agreed to do so received a Salivette (Sarstedt AG and Co, Nümbrecht, Germany) for saliva collection by regular mail. Samples were taken within 10 min after awakening on a working day, and before brushing teeth, eating, drinking, or smoking. The women were asked to record the date, time of awakening, and time of saliva collection. All samples were returned to the research site in a special envelope for biological materials by regular mail (median time between saliva collection and return: 3 days). The saliva samples were immediately stored at − 20 °C upon return. Samples that were received within 14 days after sampling and collected within 1 month after completing the second prenatal questionnaire were considered eligible for this study.

We used a previously described method to determine the cortisol concentration in the saliva samples selected [33]. At LDN Labor Diagnostika Nord GmbH and Co. KG, Nordhorn, Germany, the frozen samples were thawed before analysis and centrifuged for 5–10 min at 2000–3000 ×*g*. The concentration of cortisol in the samples was determined using the Cortisol free in Saliva ELISA Kit was used (Cortisol Saliva ELISA^free^ Kit). A total of 50 µL of the saliva sample, the standard reagent, and the control reagent was dispensed in microtiter wells. In addition, 50 µL of a cortisol-horseradish peroxidase conjugate for binding to the coated antibody, after which the wells were incubated at room temperature for 60 min. Subsequently, they were rinsed 3 times with 300 µL diluted wash solution and 200 µL of substrate solution was added. Again, the wells were incubated for 30 min at room temperature and 50 µL of stop solution was added to each well. The absorbance of each well was determined with a microtiter plate calibrated reader at 450 ± 10 nm within 15 min.

### Nested case–control design

From the PRIDE Study participants who completed the second prenatal questionnaire between April 2012 and May 2016 and provided an eligible saliva sample, we selected all cases with preterm birth (< 37 weeks of gestation), low birth weight (< 2500 g), and/or SGA (birth weight below the 10th percentile for gestational age adjusted for parity and sex) [34]. These cases were primarily identified using validated questionnaire data [35], supplemented with data from obstetric records in case of loss to follow-up. As controls, we randomly selected subjects with saliva samples from the same time period, but without any of the above-mentioned outcomes, with a 1:4 ratio for the most prevalent outcome (i.e. SGA) for optimal study power.

### Statistical analyses

Descriptive statistics were used to describe the characteristics of the women included in this nested case–control study. Using linear regression analysis, crude and adjusted β coefficients with 95% confidence intervals (CI) were estimated for the associations between maternal salivary cortisol levels and the adverse birth outcomes. These models were adjusted for potential confounders that were identified a priori by means of literature review, including maternal age (< 30, 30–34, ≥ 35 years), pre-pregnancy Body Mass Index (BMI; < 25.0 vs ≥ 25.0 kg/m^2^), parity (0 vs ≥ 1 previous birth), smoking during pregnancy, the presence of depressive symptoms based on the Edinburgh Depression Scale with a cut-off value of 10 [36, 37], gestational age at sampling, and days between sampling and freezing. As single salivary samples may suffer from intra-individual variability, however, we also categorized the maternal awakening cortisol levels using two different cut-off points from previous research based on cortisol levels in the control group: (1) dichotomized cortisol levels at the 75th percentile classifying women as having normal or elevated cortisol levels [33], and (2) trichotomized cortisol levels around the 50th and 90th percentile divided into low, moderate, and high [7]. Univariable logistic regression analyses were initially conducted to obtain crude odds ratios (OR) with 95% CIs for the associations between cortisol levels and the selected birth outcomes. Adjusted ORs were estimated from multivariable logistic regression models, including the same confounder set as the analyses on the continuous exposure.

In exploratory analyses, we stratified by infant sex to examine the potential effects that the sex of the infant might have on the associations between maternal cortisol levels and the selected birth outcomes. When < 5 cases were exposed, only crude ORs with Fisher exact 95% CIs were calculated using Episheet [38] instead of crude and adjusted ORs. All other statistical analyses were performed using SPSS version 25 for Windows (IBM Corp., Armonk, NY, USA).

## Results

**Fig. 1:**
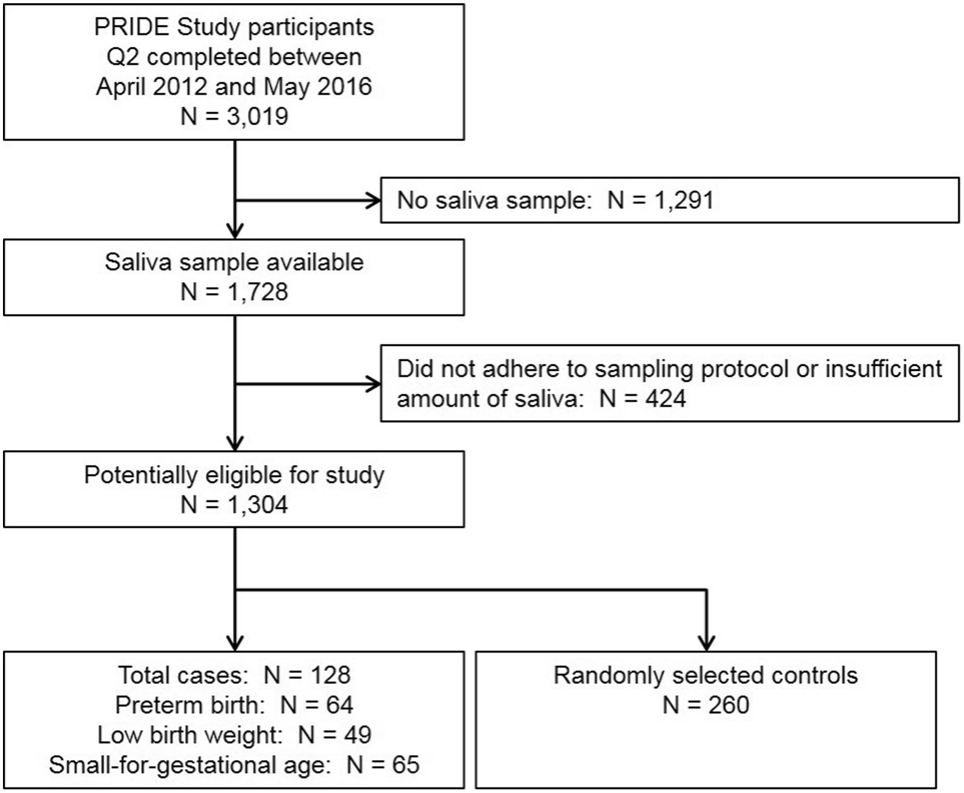
Flowchart of participation in the nested case–control study on the association between maternal awakening salivary cortisol levels and selected birth outcomes

From the 3019 PRIDE Study participants who completed the second prenatal questionnaire in the study period, 1728 (57.2%) donated a saliva sample (Fig. 1). After exclusion of women who did not adhere to the sampling protocol or donated an insufficient amount of saliva, we identified 64 cases of preterm birth, 49 cases of low birth weight, and 65 cases of SGA, 42 of which were born with more than one adverse birth outcome. In addition, we sampled 260 control infants without the selected adverse birth outcomes.

The characteristics of the women and children included in the current study are presented in Table 1. Women with one of the selected birth outcomes were slightly older and were more likely to have had preeclampsia compared with control women. In addition, women with a preterm birth or an infant with low birth weight were more likely to be primiparae than control women. The latter case group was also more likely to have used alcohol during pregnancy compared with the control group. Among women in the control group, the median cortisol level was 9.0 ng/ml (interquartile range 6.5–12.3), with the 90th percentile at 14.8 ng/ml. The median cortisol levels in the case groups were in the same order of magnitude.

**Table 1:** Maternal and pregnancy characteristics of cases of preterm birth, low birth weight, and small-for-gestational age and randomly selected control infants

Characteristic	Controls (*N* = 260)		Preterm birth (*N* = 64)		Low birth weight (*N* = 49)		SGA (*N* = 65)	
	*n*	(%)	*n*	(%)	*n*	(%)	*n*	(%)
Maternal age								
< 30 years	96	(37)	22	(34)	15	(31)	20	(31)
30–34 years	125	(48)	29	(45)	21	(43)	32	(49)
≥ 35 years	39	(15)	13	(20)	13	(27)	13	(20)
Maternal country of birth								
Netherlands	251	(97)	62	(97)	47	(96)	60	(92)
Other	9	(4)	2	(3)	2	(4)	4	(6)
Level of education								
Low/intermediate	53	(20)	10	(16)	8	(16)	11	(17)
High	207	(80)	54	(84)	41	(84)	52	(80)
Pre-pregnancy BMI^b^								
Underweight/normal weight	203	(78)	48	(75)	41	(84)	55	(85)
Overweight/obese	56	(22)	16	(25)	8	(16)	10	(15)
Parity								
0 previous births	146	(56)	51	(80)	37	(76)	34	(52)
≥ 1 previous birth	114	(44)	13	(20)	12	(25)	31	(48)
Smoking in pregnancy								
Yes	14	(5)	2	(3)	4	(8)	5	(8)
No	246	(95)	62	(97)	45	(92)	60	(92)
Alcohol use in pregnancy								
Yes	47	(18)	13	(20)	15	(31)	10	(15)
No	213	(82)	51	(80)	34	(69)	55	(85)
Depressive symptoms^c^								
Yes	30	(12)	4	(6)	6	(12)	6	(9)
No	218	(84)	57	(89)	40	(82)	56	(86)
Pregnancy complications								
Gestational diabetes	5	(2)	2	(3)	0	(0)	0	(0)
Gestational hypertension	18	(7)	3	(5)	4	(8)	6	(10)
Preeclampsia	7	(3)	5	(8)	6	(12)	6	(10)
Mean gestational week at sampling (SD)	18.8	(2.1)	18.9	(2.1)	19.2	(1.8)	19.4	(2.3)
Mean interval (minutes) between awakening and sampling (SD)	5	(3)	5	(3)	5	(3)	5	(3)
Median cortisol level (IQR)	9.0	(6.5–12.3)	9.1	(6.2–13.1)	9.3	(6.7–12.5)	9.4	(6.5–13.8)

Data from the PRIDE Study, 2012-2016^a^

*BMI* body mass index, *IQR* interquartile range, *SD* standard deviation, *SGA* small-for-gestational age

^a^Values may not add up due to missing values and rounding

^b^Classification of the National Institutes of Health

^c^Based on the Edinburgh Depression Scale [33, 34] in the second prenatal questionnaire

After correction for covariates, continuous cortisol levels were not associated with preterm birth (*β* = 0.40, 95% CI − 0.98 to 1.78), low birth weight (*β* = 0.64, 95% CI − 0.90 to 2.17), and SGA (*β* = 0.45, 95% CI − 0.92 to 1.81; Table 2). Restricting the analyses to cases with a single outcome moved the adjusted βs for preterm birth and SGA closer to the null value, whereas the adjusted β for low birth weight increased. However, this observation was based on only three cases.

**Table 2:** Associations between maternal awakening cortisol levels in mid-pregnancy and preterm birth, low birth weight, and small-for-gestational age in linear regression analyses

Outcome	*N*	Crude		Adjusted^a^	
		β	(95% CI)	β	(95% CI)
All cases					
Preterm birth	64	0.02	(− 1.34 to 1.37)	0.40	(− 0.98 to 1.78)
Low birth weight	49	0.39	(− 1.12 to 1.89)	0.64	(− 0.90 to 2.17)
Small-for-gestational age	65	0.53	(− 0.82 to 1.88)	0.45	(− 0.92 to 1.81)
Cases with single outcome					
Preterm birth	35	− 0.51	(− 2.22 to 1.20)	− 0.07	(− 1.79 to 1.64)
Low birth weight	3	1.52	(− 3.98 to 7.02)	2.05	(− 3.42 to 7.51)
Small-for-gestational age	44	0.33	(− 1.21 to 1.87)	0.29	(− 1.26 to 1.84)

Data from the PRIDE Study, 2012–2016

*CI* confidence interval

^a^Adjusted for maternal age, parity, pre-pregnancy BMI, smoking, depressive symptoms based on the Edinburgh Depression Scale, gestational age at sampling, and days between sampling and freezing

In Table 3, the crude and adjusted ORs with their 95% CIs are shown for the associations between the categorized cortisol levels and the selected birth outcomes. We did not observe associations between maternal awakening salivary cortisol levels and preterm birth and low birth weight. A high cortisol level (i.e. ≥ 90th percentile), however, seemed to be associated with an increased risk of SGA (adjusted OR 2.1; 95% CI 0.9–4.8). Restricting the analyses to cases with a single outcome yielded comparable risk estimates, with slightly higher point estimates for SGA (Table 4).

**Table 3:** Associations between awakening salivary cortisol levels in mid-pregnancy and the risk of preterm birth, low birth weight, and small-for-gestational age in logistic regression analyses.

Cortisol level	Controls		Preterm birth				Low birth weight				Small-for-gestational age			
	*n*	(%)	n	(%)	cOR (95% CI)	aOR (95% CI)^a^	*n*	(%)	cOR (95% CI)	aOR (95% CI)^a^	*n*	(%)	cOR (95% CI)	aOR (95% CI)^a^
Dichotomous														
Normal	194	(75)	46	(72)	Reference		37	(76)	Reference		45	(69)	Reference	
Elevated	66	(25)	18	(28)	1.2 (0.6–2.1)	1.4 (0.7–2.6)	12	(25)	1.0 (0.5–1.9)	1.1 (0.5–2.2)	20	(31)	1.3 (0.7–2.4)	1.3 (0.7–2.4)
Trichotomous														
Low	130	(50)	32	(50)	Reference		24	(49)	Reference		30	(46)	Reference	
Moderate	104	(40)	24	(38)	0.9 (0.5–1.7)	1.1 (0.6–2.0)	18	(37)	0.9 (0.5–1.8)	1.1 (0.5–2.2)	23	(35)	1.0 (0.5–1.7)	0.9 (0.5–1.7)
High	26	(10)	8	(13)	1.3 (0.5–3.0)	1.6 (0.6–4.4)	7	(14)	1.5 (0.6–3.7)	1.7 (0.6–4.8)	12	(19)	2.0 (0.9–4.4)	2.1 (0.9–4.8)

Data from the PRIDE Study, 2012–2016

*CI* confidence interval, *cOR* crude odds ratio, *aOR* adjusted odds ratio

^a^Adjusted for maternal age, parity, pre-pregnancy BMI, smoking, depressive symptoms based on the Edinburgh Depression Scale, gestational age at sampling, and days between sampling and freezing

**Table 4:** Associations between awakening salivary cortisol levels in mid-pregnancy and the risk of preterm birth, low birth weight, and small-for-gestational age in logistic regression analyses, restricted to cases with a single outcome.

Cortisol level	Controls		Preterm birth (*N* = 35)				Low birth weight (*N* = 3)				Small-for-gestational age (*N* = 44)			
	*n*	(%)	*n*	(%)	cOR (95% CI)	aOR (95% CI)^a^	*n*	(%)	cOR (95% CI)	aOR (95% CI)^a^	*n*	(%)	cOR (95% CI)	aOR (95% CI)^a^
Dichotomous														
Normal	194	(75)	25	(71)	Reference		2	(67)	Reference		29	(66)	Reference	
Elevated	66	(25)	10	(29)	1.2 (0.5–2.6)	1.3 (0.6–3.1)	1	(33)	–	–	15	(34)	1.5 (0.8–3.0)	1.6 (0.8–3.2)
Trichotomous														
Low	130	(50)	18	(51)	Reference		0	(0)	Reference		18	(41)	Reference	
Moderate	104	(40)	13	(37)	0.9 (0.4–1.9)	1.0 (0.5–2.3)	3	(100)	–	–	18	(41)	1.3 (0.6–2.5)	1.2 (0.6–2.4)
High	26	(10)	4	(11)	1.1 (0.3–3.6)	1.4 (0.4–5.3)	0	(0)	–	–	8	(18)	2.2 (0.9–5.7)	2.6 (0.9–6.9)

Data from the PRIDE Study, 2012–2016

*CI* confidence interval, *cOR* crude odds ratio *aOR* adjusted odds ratio

^a^Adjusted for maternal age, parity, pre-pregnancy BMI, smoking, depressive symptoms based on the Edinburgh Depression Scale, gestational age at sampling, and days between sampling and freezing

After stratification by infant sex in the exploratory analyses, we did not observe associations between continuous maternal cortisol levels and preterm birth (boys: adjusted *β* = -0.48 [95% CI − 2.32 to 1.36], girls: adjusted *β* = 0.73 [95% CI − 1.36 to 2.83]), low birth weight (boys: adjusted *β* = 0.83 [95% CI − 1.21 to 2.88], girls: adjusted *β* = 1.00 [95% CI − 1.20 to 3.20]), and SGA (boys: adjusted *β* = 0.08 [95% CI − 1.60 to 1.76], girls: adjusted *β* = 1.31 [95% CI − 0.80 to 3.43]). The results of the stratified analyses on the categorical exposure variables are shown in Table 5. Among women who delivered a girl, a high cortisol level was associated with preterm birth (adjusted OR 4.3; 95% CI 1.1–16.9) and SGA (adjusted OR 3.7; 95% CI 1.1–11.9). We did not observe any associations among women who delivered a boy.

**Table 5:** Results of the exploratory analyses on associations between awakening salivary cortisol levels in mid-pregnancy and the risk of preterm birth, low birth weight, and small-for-gestational age, stratified for infant sex.

Cortisol level	Controls		Preterm birth				Low birth weight				Small-for-gestational age			
	*n*	(%)	*n*	(%)	cOR (95% CI)^a^	aOR (95% CI)^b^	*n*	(%)	cOR (95% CI)^a^	aOR (95% CI)^b^	*n*	(%)	cOR (95% CI)^a^	aOR (95% CI)^b^
Infant sex: male														
Dichotomous														
Normal	88	(73)	24	(83)	Reference		17	(81)	Reference		25	(74)	Reference	
Elevated	32	(27)	5	(17)	0.6 (0.2–1.6)	0.5 (0.2–1.7)	4	(19)	0.6 (0.1–2.2)	–	9	(27)	1.0 (0.4–2.3)	1.0 (0.4–2.4)
Trichotomous														
Low	60	(50)	14	(48)	Reference		9	(43)	Reference		16	(47)	Reference	
Moderate	49	(41)	14	(48)	1.2 (0.5–2.8)	1.2 (0.5–2.9)	10	(48)	1.4 (0.5–3.6)	1.4 (0.5–4.0)	14	(41)	1.1 (0.5–2.4)	1.0 (0.5–2.4)
High	11	(9)	1	(3)	–	–	2	(10)	1.2 (0.1–7.1)	–	4	(12)	1.4 (0.3–5.4)	–
Infant sex: female														
Dichotomous														
Normal	106	(76)	22	(63)	Reference		20	(71)	Reference		20	(65)	Reference	
Elevated	34	(24)	13	(37)	1.8 (0.8–4.0)	2.4 (1.0–6.0)	8	(29)	1.2 (0.5–3.1)	1.8 (0.6–5.0)	11	(36)	1.7 (0.7–3.9)	2.1 (0.8–5.1)
Trichotomous														
Low	70	(50)	18	(51)	Reference		15	(54)	Reference		14	(45)	Reference	
Moderate	55	(39)	10	(29)	0.7 (0.3–1.7)	0.7 (0.3–1.9)	8	(29)	0.7 (0.3–1.7)	0.8 (0.3–2.5)	9	(29)	0.8 (0.3–2.0)	0.7 (0.3–2.0)
High	15	(11)	7	(20)	1.8 (0.6–5.1)	4.3 (1.1–16.9)	5	(18)	1.6 (0.5–4.9)	3.1 (0.7–13.3)	8	(26)	2.7 (1.0–7.5)	3.7 (1.1–11.9)

Data from the PRIDE Study, 2012–2016

*CI* confidence interval, *cOR* crude odds ratio *aOR* adjusted odds ratio

^a^For exposure groups with < 5 exposed cases, presented ORs are crude ORs with Fisher exact 95% CIs

^b^Adjusted for maternal age, parity, pre-pregnancy BMI, smoking, depressive symptoms based on the Edinburgh Depression Scale, gestational age at sampling, and days between sampling and freezing

## Discussion

In this study, we did not identify strong associations between mid-pregnancy maternal awakening salivary cortisol levels and the occurrence of preterm birth and low birth weight. However, a possible association was observed between high cortisol levels in mid-pregnancy (i.e. ≥ 90th percentile) and SGA. In the exploratory analyses, this risk of SGA was only increased among female infants, just as the risk of preterm birth, but these analyses relied on small sample sizes, resulting in unstable effect estimates.

Although cortisol has repeatedly been hypothesized as one of the biological intermediates linking prenatal psychosocial stress to adverse birth outcomes [6–8], studies examining the associations between maternal cortisol levels during pregnancy and adverse birth outcomes showed inconsistent results. Reasons for these inconsistencies include differences in biomarkers for cortisol, the timing and extent of biomarker collection throughout the day, and the variability in the timing of assessment during gestation, measuring in early, mid or late pregnancy [39, 40]. Some, mostly small, studies found associations between elevated maternal cortisol levels and the risk of preterm birth in the entire population or in subgroups [19, 41, 42], although publication bias may be an issue here. Other published studies did not observe associations between maternal cortisol levels during pregnancy and preterm birth [20, 43, 44], in line with our results. In a recent meta-analysis among 1606 maternal–fetal dyads [21], a negative association was observed between maternal salivary cortisol and infant birth weight. However, the risk of low birth weight, a clinically relevant outcome measure, was not assessed. In a large prospective cohort study of 2810 women not included in this meta-analysis, no associations were observed between elevated serum cortisol levels measured in early pregnancy and offspring birth weight [7]. Furthermore, cortisol was not associated with self-reported measures of psychological functioning among pregnant women in several studies [11–13, 23], making it unlikely that cortisol can be classified as biomarker of the mechanistic pathway linking maternal psychosocial problems to preterm birth and birth weight according to the ‘Meet in the Middle’ approach. Alternative markers of stress related to the autonomous nervous system and inflammatory response system may also play a more prominent role in the association between maternal prenatal stress and these birth outcomes [28, 45], and should be taken into account in future studies.

In the same prospective cohort study of 2810 women, an association was observed between high morning cortisol levels (≥ 90th percentile) and an increased risk of SGA, concordant with our findings. Several potential pathways underlying the associations between elevated maternal cortisol levels and adverse birth outcomes have been suggested, including dysregulation of the fetal autonomic nervous system [6]﻿, vasoconstriction of the uterine artery resulting in reduced uterine blood flow [17], and stimulation of the production and release of placental CRH [18]. Specifically for SGA, an association with disturbed expression and/or activity of placental 11β-hydroxysteroid dehydrogenase type 2 (11β-HSD2), an enzyme that protects the fetus from high levels of maternal cortisol, has been observed [46–50]. Interestingly, decreased functioning of 11β-HSD2 in SGA infants has only been observed in pregnancies with female fetuses [51]. Recent findings on sex-specific differences in systemic glucocorticoid imbalance [52] also strengthen the plausibility of our finding that elevated maternal salivary cortisol increases the risk of SGA among female fetuses only. Likewise, previous research indicated that the effects of depression during pregnancy could have a different impact on the two sexes [53].

Strengths of this study include the nested case–control design with embedding in a large prospective cohort study. Due to this design, we were able to include a relatively large number of cases compared to previous smaller studies [6, 23, 41]. This made it possible to perform stratified analyses for infant sex, which could modify associations between increased cortisol levels and adverse birth outcomes. The design also led to the availability of prospectively collected information on many maternal characteristics, which were used to adjust for potential confounding effects on the associations of interest. Previous studies showed that maternal characteristics, such as parity, smoking behavior, and BMI, could have an influence on maternal cortisol levels [7, 54, 55]. Adjustment for these factors was often not completely or not at all possible in previous studies, resulting in potentially biased effect estimates due to residual confounding.

A limitation of the current study is the collection of only a single awakening saliva sample to determine the cortisol level, whereas multiple measurements during the day or on consecutive days might be preferred. In a previous study validating this approach, however, we showed that a single awakening salivary sample could reliably distinguish between women having normal and elevated cortisol levels [33]. Nevertheless, we were unable to assess the cortisol awakening response and exposure patterns or trajectories throughout pregnancy with a single sample. As the saliva sample was collected in mid-pregnancy, we could not examine the associations between adverse birth outcomes and fetal exposure to elevated cortisol levels in early or late pregnancy. The critical exposure windows for the risks of preterm birth, low birth weight, and SGA are currently unknown [7, 56], but most previous studies collected the biomarkers for cortisol around gestational week 20, comparable to our study. By performing stratified exploratory analyses, we obtained more insight into the potential sex-dependent effects of elevated maternal cortisol levels. Due to small numbers of exposed cases in some of these exploratory analyses, however, we cannot draw firm conclusions on the sex-specific associations. Therefore, these analyses should be repeated in larger studies.

## Conclusion

The results of this nested case–control study showed no indications that maternal cortisol levels in mid-pregnancy are strongly associated with increased risks of preterm birth and low birth weight, but they may be associated with an increased risk of SGA. Infant sex seemed to influence this association. These findings help to understand the etiology and the onset of the occurrence of adverse birth outcomes and contribute to growing knowledge of the complex biological pathways linking maternal psychosocial problems to adverse birth outcomes.
